# Supplementary material for: Patterns of Intron Gain and Loss in Fungi
Source: PLoS Biol. 2004 Nov 30;2(12):e422. doi: 10.1371/journal.pbio.0020422 (PMC532390; doi:10.1371/journal.pbio.0020422)
Supplement: Table S1 — Also available at http://genes.mit.edu/NielsenEtAl/. (4.3 MB ZIP). [file pbio.0020422.st001.zip › NielsenEtAl/html/30.html]

AN1526.1.NCU00966.1.MG10831.1.FG05601.1


```
 CLUSTAL W (1.82) Multiple Sequence Alignments - Introns Inserted


Sequence 1: NCU00966.1	259 aa
Sequence 2: FG05601.1	260 aa
Sequence 3: MG10831.1	261 aa
Sequence 4: AN1526.1	266 aa
Alignment Length: 266 aa
Number Identitical Residues: 133 aa
Alignment Score (without introns) 6708


MG10831.1 	MAAQVPVQTIHRDPQLF2YWILGPITVVM0ILTGMLRHYAAVLMAVAPKK-VDAKAMREQ
NCU00966.1	-MAQVPVQTIHRDPQLF2YWILFPITIVM0ILTGVLRHYASVLMQSAPKK-LDQKAIREQ
FG05601.1 	-MAQVPVQTLHRDPQLF2YWILIPITIVM~VLTGVLRHYVSVLMATAPKK-QDEKTMREQ
AN1526.1  	MALQGVEQTILRDPALF2YWILIPISVVM0ILTGMLRHYATILMNSPPKPPATLAESRER
          	   *   **: *** ** **** **::** :***:****.::**  .** .      **:

MG10831.1 	RSLLHGINVRSNY-HVLSKRSFNTRRDTLSSAYESGAFLKAPESRGQAPPNPMTDPNAME
NCU00966.1	RSLLHGINLRGNF-HALSKKSFATRRDALVTAFESGAYLKDPERAGQGQPNPLSDPSSME
FG05601.1 	RSLARGVALRSNH-HVLSQKAFESRRDILTTNYEAGTYLKEPDRKGQPPANPLSDPGAMD
AN1526.1  	LSLFRAVNLRNHAPAVLSKEAFEMRKNYLVTGFQTGAFLKDPNSRGQPPANPMSDPAAME
          	 ** :.: :*.: . .**:.:*  *:: * : :::*::** *:  **  .**::** :*:

MG10831.1 	GMMGMMKNNMAMIVPNTLIMSWINAFFSGYVIM1KLPFPLTVKFKSMLQAGVATRDMDPR
NCU00966.1	GMMGMMKNQMAMIIPNTLIMSWINAFFSGYVIM1KLPFPLTIKFKSMLQAGVMTKDMDPR
FG05601.1 	GMMGMMKNNMAMIIPNTLIMSWINAFFSGYVIM1KLPFPITIKFKSMLQAGVQTREMDPR
AN1526.1  	GMMGMMKGNMMMMIPQTLIMSWINAFFSGFVIL1KLPFPLTIRFKSMLQSGVMTRDLDVR
          	*******.:* *::*:*************:**: *****:*::******:** *:::* *

MG10831.1 	WMSSISWYFLCIFGLQFVFNFLLGSDNA1ANQMAQQMGQMGPQA-PQMFGPGVDPDKQFL
NCU00966.1	WMSSISWYFLCIFGLQSVFNFLLGSDNA1ASQMAQQMGGMGAAP-QMFG-PGVDPDKQFK
FG05601.1 	WMSSISWYFLCIFGLQFVYVFLLGSDNA1ASQVAQQMQAQQMPN-PMMGGPGQDPHKQFV
AN1526.1  	WVSSLSWYFLNLFGLQSVFGFILGSDNA1ANHMAQQMGPMGPAAGANPFQPGQDPHKMYL
          	*:**:***** :**** *: *:****** *.::****       .     ** **.* : 

MG10831.1 	AEAENLAVIEHYSVLDDVEDRLLERLGA--
NCU00966.1	AEAENLAVIEHYSVLDNVEERLLEGITV--
FG05601.1 	AEVENLNVVEHYSVLDNVEERLLAGIKS--
AN1526.1  	NEAENLEVFEHFSILDGIEDRVLRKYGAAQ
          	 *.*** *.**:*:**.:*:*:*     :.
```
